# Close Adherence to a Mediterranean Diet during Pregnancy Decreases Childhood Overweight/Obesity: A Prospective Study

**DOI:** 10.3390/nu16040532

**Published:** 2024-02-14

**Authors:** Andrés Díaz-López, Laura Rodríguez Espelt, Susana Abajo, Victoria Arija

**Affiliations:** 1 Nutrition and Mental Health (NUTRISAM) Research Group, Universitat Rovira i Virgili (URV), 43204 Reus, Spain; andres.diaz@urv.cat (A.D.-L.); laura.rodrigueze@alumni.urv.cat (L.R.E.); 2 Institut d’Investigació Sanitària Pere Virgili (IISPV), 43005 Tarragona, Spain; 3 Sexual and Reproductive Healthcare Service of Reus-Tarragona, Institut Català de la Salut, Generalitat de Catalunya, 43003 Tarragona, Spain; sabajo.tgn.ics@gencat.cat; 4 Atención Primaria, Centro de Salud Embajadores, Dirección Asistencial Noroeste, 28012 Madrid, Spain; 5 Collaborative Group on Lifestyles, Nutrition, and Tobacco (CENIT), Tarragona-Reus Research Support Unit, Jordi Gol Primary Care Research Institute, 43202 Reus, Spain

**Keywords:** Mediterranean diet, lifestyles, pregnancy, childhood overweight, childhood obesity, ECLIPSES

## Abstract

The study of dietary patterns during pregnancy may be of great importance for determining the potential risk of obesity in childhood. We assessed the prospective association between maternal adherence to the Mediterranean diet (MedDiet) during pregnancy and risk of childhood overweight/obesity at 4 years. This prospective analysis involved 272 mother–child pairs from the ECLIPSES study. Maternal diet during pregnancy was assessed using a validated 45-item food-frequency questionnaire and a relative whole-pregnancy MedDiet score (rMedDiet) was calculated. The children’s weight and height were measured at the age of 4. Primary outcome was childhood overweight/obesity based on age- and-sex-specific BMI z-score > 85th percentile using the WHO child growth standards. Mean maternal rMedDiet score in pregnancy was 9.8 (±standard deviation 2.3) and 25.7% of the children were overweight/obese. Significant differences in anthropometric measurements (weight, height, and BMI) were found according to sex, with higher scores for boys. After controlling for potential confounders, greater maternal adherence to rMedDiet during pregnancy was associated with a lower risk of childhood overweight/obesity, highest vs. lowest quartile (OR = 0.34, 95% CI: 0.12–0.90; *p*-trend 0.037). Similar trends regarding this association (per 1-point increase rMedDiet score) were observed after stratification by advanced maternal age, maternal early pregnancy BMI, education, socioeconomic status, smoking, and gestational weight gain. Our findings suggest that closer adherence to the MedDiet during pregnancy may protect against the risk of offspring overweight/obesity at 4 years. Further research is needed to explore whether associations persist across the life course.

## 1. Introduction

The current childhood overweight/obesity epidemic represents a global public health problem of the first magnitude, affecting 42 million children under 5 years of age [1]. According to Aranceta-Bartrina et al. [2] in a study conducted in the Spanish population in 2015, the prevalence of overweight and obesity was 23.9% and 15.9% in children aged 3 and 8 years, respectively. This epidemic has serious health consequences, including psychological comorbidities such as depression, anxiety, low self-esteem, and a series of emotional and behavioral disorders in childhood [1]. Children who are overweight and obese not only have a higher likelihood of obesity in adolescence and adulthood but are also more prone to long-term cardiovascular morbidity [3] and cancer, which can lead to disability and premature death [4]. Since childhood overweight or obesity is largely preventable, identifying early determinants to mitigate this condition and its negative health effects has become a top public health priority [5].

In this context, the WHO Report of the Commission on Ending Childhood Obesity reiterates the significance of appropriate antenatal nutritional balance in terms of quantity and quality as a crucial modifiable factor in preventing childhood obesity [6]. Close maternal adherence to a Mediterranean-style diet (MedDiet) during gestation could therefore be a promising strategy.

The MedDiet is believed to be one of the healthiest eating patterns and to guarantee a better supply of nutrients. This traditional eating pattern is characterized by a high intake of plant foods (fruits and vegetables, nuts and legumes, whole grain products, and olive oil), a moderate intake of fish, and only a small intake of red and processed meats. Several observational and intervention studies provide evidence to support the role of the MedDiet in the prevention and management of obesity, type-2 diabetes, and the metabolic syndrome in adults [7,8].

Recent research also suggests that it has a protective effect against childhood overweight/obesity [9,10,11]. In pregnancy, closer maternal adherence to the MedDiet has been inversely associated with potential risk factors for childhood obesity in early life, such as preterm delivery [12], fetal growth restriction [13], and small-for-gestational-age (SGA) infants at birth [14]. Moreover, lower MedDiet scores in early pregnancy have been reported to negatively impact insulin resistance markers at birth [15], potentially contributing to later childhood obesity.

However, evidence from both Mediterranean and non-Mediterranean populations examining the impact of adhering to the MedDiet during pregnancy on offspring’s body composition in infancy or the risk of childhood overweight and/or obesity is scarce and, due to the varied findings in studies, unconvincing [16,17,18,19,20,21]. The lack of consensus among previous studies may be due to variations in adherence to this dietary pattern owing to differences in cultural, sociodemographic, or lifestyle aspects as well as to the methodologies employed to evaluate the MedDiet score, the assessment period during pregnancy, the characteristics of the population, and the time of follow-up.

It is also worth noting that previous studies on this topic have rarely reported subgroup analyses for relevant maternal obesogenic confounders such as higher weight status, excessive gestational weight gain, poorer education, lower socioeconomic status, and smoking—all of which, in turn, have also been linked to poor adherence to such eating pattern. In light of the above, examining whether a prenatal MedDiet affects childhood overweight/obesity and whether effects depend on these maternal behaviors may provide valuable insights for future family-focused efforts aimed at preventing child obesity.

The aims of the present study, framed within the Mediterranean ECLIPSES cohort of healthy women and their respective children, are to prospectively assess the association between maternal adherence to the MedDiet during pregnancy and the risk of childhood overweight/obesity at the age of 4, and to determine whether this association varies depending on maternal environmental factors.

## 2. Materials and Methods

### 2.1. Study Design and Participants

This study corresponds to longitudinal analysis based on a subsample of healthy pregnant women who participated in the ECLIPSES (Ensayo CLInico Para Suplementar con hierro a EmbarazadaS) study, as well as data from their children at the age of 4. Briefly, ECLIPSES is a community randomized controlled trial conducted in the province of Tarragona (Catalonia, Spain) between 2013 and 2017. The study aimed to evaluate the effectiveness of prenatal iron supplements during pregnancy in different doses adjusted for the initial levels of hemoglobin levels on maternal iron status at the end of pregnancy [22]. Details of the study’s protocol have been described elsewhere [22]. A total of 791 pregnant women were recruited during the first prenatal visit (before 12 weeks of gestation) by midwives at their primary care centers. Eligible participants were healthy adult women older than 18 years with ≤12 weeks of gestation. Further details of the inclusion/exclusion criteria can be found elsewhere [22].

**Figure 1:**
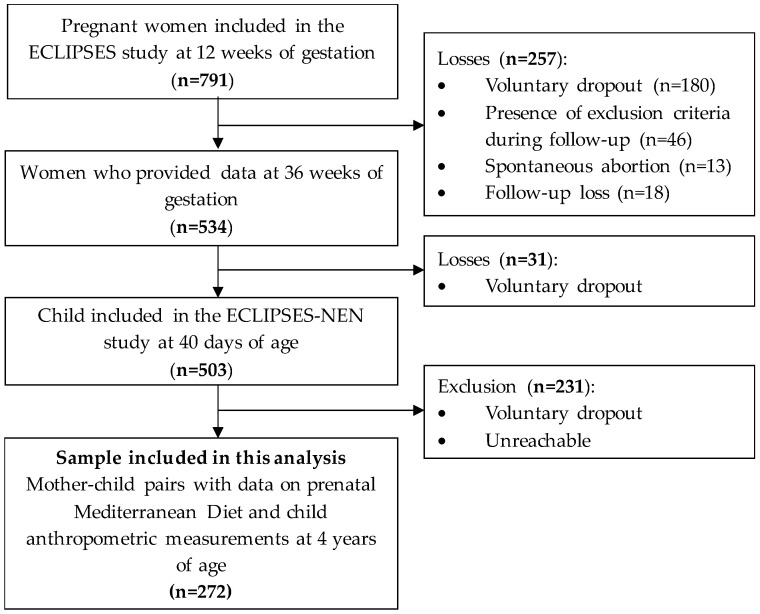
Flowchart of the study.

Of all the pregnant women initially recruited, 272 mother–child pairs returned for a study visit at 4 years post-delivery and were considered in the present prospective analyses (Figure 1). It is worth noting that the greatest dropout rate mainly occurred after the post-intervention period, often due to either voluntary withdrawal or being unreachable.

The ECLIPSES trial is registered at both the ClinicalTrials.gov (identification number NCT03196882) and the EU Clinical Trials Register (EUCTR-2012-005480-28). This study was approved by the Ethical Committee of the Pere Virgili Institute for Health Research (IISPV). All participants signed an informed consent form. This study complies with the tenets of the Declaration of Helsinki.

### 2.2. Dietary Assessment

Maternal dietary intake was assessed using a food-frequency questionnaire (FFQ), which had been validated in our population [23], at weeks 12, 24, and 36 of gestation. The questionnaire asked 45 food-intake questions by week and month. Consumption in grams per day of each item was calculated by applying the average ration of consumption usually observed in our population [23]. Total daily intake of energy was estimated using the REGAL food table [24].

From the FFQ, we calculated a relative MedDiet score (rMedDiet) based on the intake of nine nutritional components that capture the essence of the traditional MedDiet—a method previously used in our published paper [14,25]. Each rMedDiet component (apart from alcohol) was expressed in grams per 1000 kcal/day and divided by tertiles of dietary intake. Values of 0, 1, or 2 points were assigned to each tertile. Six of the nine components (vegetables, fruits and nuts, legumes, cereals, fresh fish, and olive oil) scored positively, while the scoring was reversed for two components (meat/meat products and dairy products). Alcohol, which is considered harmful during the gestation period, was assigned 0 points for women who consumed it and 2 points for women who did not. The possible scores assigned to each pregnant woman ranged from 0 to 18 points, with larger values indicating closer adherence to the MedDiet and therefore a higher quality of diet. For the purposes of analysis, we constructed the rMedDiet score for the whole gestation period by averaging the rMedDiet scores from the first, second, and third trimesters of pregnancy. Since there are no pre-established cut-off points for the pregnant population, we categorized the score into quartiles for this analysis.

### 2.3. Outcome

Children were followed up with at 4 years of age at healthcare centers, and anthropometric measurements were collected in person. Weight was measured using the TANITA digital scale (Body Composition Analyzer TANITA BC-418, Tanita Corporation of America, Inc., Arlington Heights, IL, USA). Height measurements were taken with a portable stadiometer (SECA 222^®^, Hamburg, Germany) ranging between 0 and 200 cm and with 1 mm precision using the Frankfurt standard. Using the child weight and height measures at 4 years of age, BMI (in kg/m^2^) was calculated and subsequently converted to z-scores using the WHO child growth standards for 0–5 years [26]. The main outcome of interest for the present study was childhood overweight and/or obesity at 4 years, which was defined as age- and sex-specific BMI z-score > 85th percentile [27].

### 2.4. Other Maternal and Child Covariates

Baseline maternal characteristics were collected from questionnaires during a face-to-face interview at recruitment.

These included medical and sociodemographic data, such as maternal age (years), socioeconomic and educational level, and other lifestyle habits (maternal smoking [yes, no], alcohol consumption [yes, no], and physical activity). Family socioeconomic status (SES) was calculated by combining information on occupational status, classified in accordance with the Catalan classification of occupations (CCO-2011) [28], and educational level. It was then classified as low, middle, or high. The women’s educational level was divided into three groups: low (primary school or less), medium (secondary studies), and high (university studies or more).

Physical activity levels were determined using the short version of the International Physical Activity Questionnaire (IPAQ) [29]. Following the IPAQ scoring protocol [29], the IPAQ responses were converted to total Metabolic Equivalent Task minutes per week (METs-min/wk) based on summation of the duration (in minutes) and frequency (days) of walking, moderate-intensity and vigorous-intensity activity, over the preceding seven-day period.

Maternal anthropometric measurements including height (cm) and weight (kg) were assessed at enrollment and during pregnancy. Based on BMI classification standards [30], women were categorized as normal weight (BMI 18.5–<25 kg/m^2^), overweight (BMI 25–<30 kg/m^2^) or obese (BMI ≥ 30 kg/m^2^) in the first trimester. Total gestational weight gain (GWG) was calculated as the difference between the maternal weight measured at the third- and first trimester visits. Considering initial BMI, GWG was categorized as insufficient, adequate, or excessive according to the recommendations of the Institute of Medicine (IOM) published in 2009 [31]. An adequate GWG corresponds to between 11.5, and 16 kg for an initial normal weight, between 7 and 11.5 kg for an initial overweight, and between 5 and 9 kg for an initial obesity. Values below or above adequate GWG were considered insufficient or excessive GWG, respectively [31].

In addition to gestational age at birth (weeks), type of delivery (vaginal, cesarean [any]), and sex, also included was information on the child’s anthropometric measurements at birth (weight and length) as they were considered potentially confounding.

### 2.5. Statistical Analysis

Descriptive data are presented as means (±SD) for quantitative variables and the number (%) for categorical variables. Differences in participant characteristics by categories of weight status of children at 4 years of age, sex, or quartiles of maternal rMedDiet total score during pregnancy were tested using chi-square, T-Student, or ANOVA test, as appropriate.

Logistic regression analysis was used to explore the associations between maternal DietMed score (as a continuous variable and quartiles) during pregnancy and childhood overweight/obesity at 4 years. We fitted a crude univariate model and, based on existing literature, constructed a multivariable model that was adjusted for the following potential confounders: maternal age [<25 (ref.), 25–29, ≥30 years], initial BMI [normal (BMI < 25 kg/m^2^) (ref.), overweight (BMI 25–29.9 kg/m^2^), obesity (BMI ≥ 30 kg/m^2^)], weight gain during pregnancy [adequate (ref.), insufficient, excessive], educational level [low (primary school or less)/medium (secondary studies) (ref.), high (university students)], SES [low/medium (ref.), high], smoking status [no (ref.), yes], mode of delivery [normal vaginal (ref.), cesarean (any)], gestational age at delivery (weeks), sex of the child (male, female), birth weight (g), and age of the child (years) at the 4-year visit. Results are expressed as odds ratio (OR) and 95% confidence intervals (CIs). For trend analysis, logistic regression analysis was repeated, with maternal adherence to the DietMed tertiles giving integer values. Analyses were conducted for the whole population since no statistical interaction was found according to the sex of the children (*p* = 0.44).

We also conducted stratified analyses to assess potential effect modification by selected subgroups: maternal age (<30, ≥30 years), early pregnancy BMI (normal weight, excess weight), GWG (insufficient, adequate, excessive), education (primary school or less/secondary, university), SES (low/middle, high), and smoking (yes, no). Multivariable-adjusted OR (95% CIs) per one-point increase in maternal rMedDiet score is also presented. Effect modification was considered statistically significant if the interaction term p-value was <0.05. Interactions were tested with the likelihood ratio tests, which involved comparing models with and without cross-product terms. Statistical analyses were performed with STATA version 15 (StataCorp LP, CollegeStation, TX, USA). *p*-values < 0.05 were defined as statistically significant.

## 3. Results

The prevalence of overweight/obesity in the children in our study at age 4 was 25.7%, with a greater prevalence observed in boys than in girls (63% vs. 37%, *p* = 0.019). The characteristics of the participants by weight status of the children are shown in Table 1. Overall, the mean age of the mothers was 31.6 ± 4.6 years and 70% of them were over 30 years old. Their mean initial BMI was 25.1 ± 4.4 kg/m^2^ and roughly 42% of the women were stratified as overweight or obese with BMI ≥ 25.0 kg/m^2^. Most mothers (86%) were Spanish, 44% had a university education, 22% were of a high SES, and 17% smoked during pregnancy. The children of mothers who were under 30 years of age, were obese (BMI ≥ 30 kg/m^2^), exceeded the criteria for GWG, or reported smoking during pregnancy were more likely to be overweight/obese at 4 years of age. Conversely, overweight/obesity was less frequent among children of mothers who had a university education, were of a higher SES, and had higher rMedDiet scores during pregnancy (Table 1).

The mean weight and BMI of the children at the 4-year visit were 18.1 ± 3.2 kg and 16.0 ± 2.1 kg/m^2^, respectively (Table 1). In general, boys had higher values for weight (18.5 ± 3.9 vs. 17.4 ± 2.2 kg, *p* = 0.005), BMI (16.4 ± 2.5 vs. 15.6 ± 1.5, *p* = 0.002), weight-for-age z-score (0.48 ± 1.34 vs. 0.14 ± 0.89, *p* = 0.015), and BMI z-score (0.69 ± 1.60 vs. 0.20 ± 0.99, *p* = 0.003) than girls.

**Figure 2:**
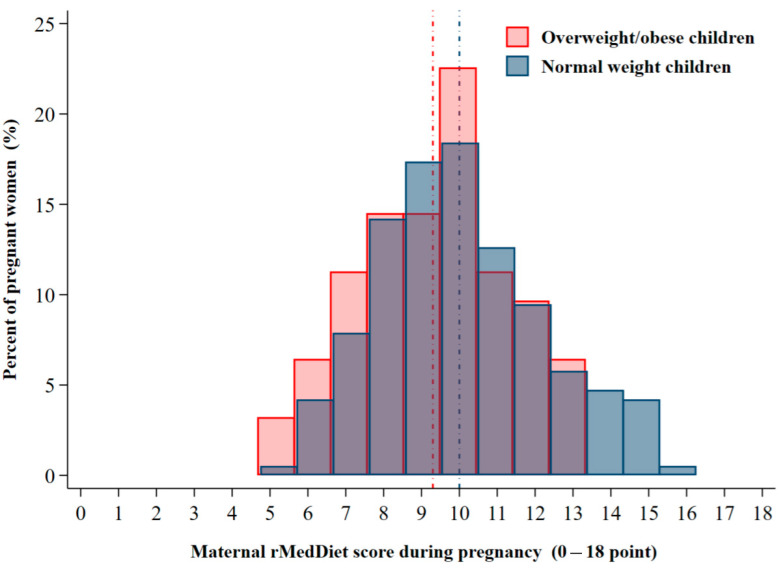
Frequency distribution of the maternal rMedDiet score during pregnancy in women whose children are of normal weight (blue bar; the vertical blue line represents the mean rMedDiet score=10.0 points) and women whose children are overweight/obese (red bar; the vertical red line represents the mean rMedDiet score=9.2 points) at 4 years of age.

The mean (SD) maternal rMedDiet score during pregnancy was 9.8 (2.3) points. Figure 2 shows the frequency distribution of the rMedDiet scores for women whose children were of normal weight and for women whose children were overweight/obese at age 4. In our sample, 29.0% of mothers were classified as being of low adherence to the rMedDiet (lowest quartile), while 22.6% were classified as being of high adherence (highest quartile).

Table 2 shows the odds ratios for the prevalence of overweight/obesity at 4 years of age according to maternal rMedDiet adherence (continuous and quartile scores) during pregnancy. After adjustment for potential confounders, each one-point increment in the maternal MedDiet total scale was associated with a 19% lower risk of childhood overweight/obesity at 4 years of age (OR: 0.81; 95% CI: 0.68–0.95; *p* = 0.010). Similarly, children of mothers with the closest adherence to the rMedDiet had lower odds for overweight/obesity than those of mothers with the lowest adherence (ORQ4 vs. Q1:0.34; 95% CI: 0.12–0.90, *p*-trend = 0.037). In the multivariable model, we also found that smoking (OR: 2.53; 95% CI: 1.10–5.83, *p* = 0.029), starting pregnancy while overweight (OR: 2.47; 95% CI: 1.04–5.87, *p* = 0.041) or obese (OR: 2.64; 95% CI: 1.01–6.93, *p* = 0.045), and excessive GWG (OR: 2.90; 95% CI: 1.26–6.69, *p* = 0.012) were all markedly associated with overweight/obesity at 4 years of age.

**Figure 3:**
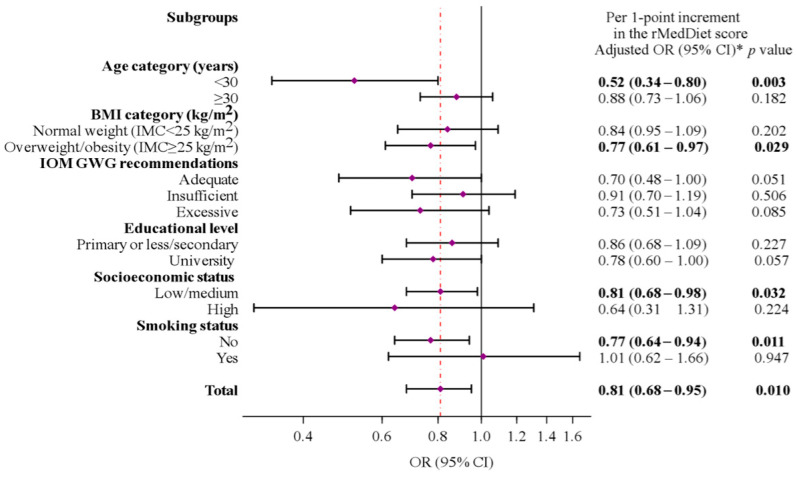
Multivariable-adjusted ORs (95% CIs) of childhood overweight/obesity at 4 years of age associated with a 1-point increase in maternal rMedDiet score during pregnancy for selected subgroups. Abbreviations: BMI, body mass index; GWG, gestational weight gain; IOM, Institute of Medicine. * Models were mutually adjusted for maternal age [<25 (ref.), 25–29, ≥30 years], initial BMI [normal (BMI < 25 kg/m^2^) (ref.), overweight (BMI 25–29.9 kg/m^2^), obesity (BMI ≥ 30 kg/m^2^)], weight gain during pregnancy [adequate (ref.), insufficient, excessive], educational level [low (primary school or less)/medium (secondary studies) (ref.), high (university students)], SES [low/medium (ref.), high], smoking status [no (ref.), yes], mode of delivery [normal vaginal (ref.), cesarean (any)], gestational age at delivery (weeks), sex of the child (male, female), birth weight (g), and age of the child (years) at the 4-year visit, except for the variables used as subgroup variables in each case. The diamonds represent OR and the whisker plots represent 95% CIs. The vertical line (red) represents the overall OR.

In stratified analyses, the protective effect of the rMedDiet (per one-point increment) on childhood overweight/obesity appeared to be more evident among pregnant women who were aged under 30, were overweight/obese at early pregnancy (BMI ≥ 25 kg/m^2^), were non-smokers, or belonged to a lower-middle social class (Figure 3). However, we observed no effect modification for these or other associations (all *p*-values for interactions were ≥0.15).

## 4. Discussion

This prospective study was conducted in a Mediterranean Spanish population of pregnant women and their children assessed at 4 years of age. We found that, irrespective of important confounders, higher maternal whole-pregnancy diet quality, as evidenced by a closer adherence to the rMedDiet pattern, was associated with lower odds for overweight/obesity in 4-year-old children. This association appeared to be more evident among pregnant women who were under 30, overweight/obese, non-smokers, or of a low-to-middle SES. Our results showed a prevalence of overweight/obesity of 25.7% in 4-year-old preschool children. This figure is in agreement with published data from 27 European countries that estimate a global prevalence of overweight/obesity in children aged from 2 to 7 years between 2006 and 2016 of 18% (23% in Spain) [32]. Similar rates of childhood overweight/obesity in Spanish preschoolers (21.4 to 34.8%) have been shown in other populations [2,17,33,34]. These results, together with the negative psychological, social, and health consequences of overweight/obesity in preschool ages, suggest that earlier primary prevention of childhood excess weight should be a major public health priority.

Our research adds to accumulating evidence that stresses the potentially important role of maternal dietary quality during pregnancy on the early life health of offspring [35]. It also suggests that modifying maternal eating patterns toward a Mediterranean-type diet could be a key strategy for preventing childhood overweight/obesity. Indeed, a large body of evidence has linked greater adherence to the MedDiet during pregnancy with multiple health benefits for mother and child [36,37], including a reduced likelihood of gestational diabetes and weight gain in mothers [38], a lower risk of SGA in infants [14], and protection against cardiometabolic disorders during childhood [16]. However, the relationship between prenatal maternal adherence to this healthy dietary pattern and the risk of early childhood overweight/obesity has been largely underexplored. The limited prior research that has been conducted on this topic mostly aligns with our findings [16,17,18,19,20,21], though not entirely [16,21].

In line with our results, a recent U.S. study from the NEST (Newborn Epigenetics STudy) cohort found that greater maternal adherence to a Mediterranean-type diet during early gestation was associated with reduced child weight-for-height percentiles at 3–8 years of age [20]. Likewise, results from the U.S. Project Viva cohort also revealed a significant inverse association between maternal first-and-second-trimester adherence to the MedDiet and child BMI z-scores as a measure of general adiposity in early (3 years) and mid-childhood (7–10 years) [19]. Another study using data pooled from both the Project Viva and the Rhea cohorts showed that in U.S. (median 7.7 years) and Greek (median 4.2 years) children, a greater adherence to the MedDiet in early pregnancy was associated with lower early childhood adiposity levels as reflected by lower BMI and lower abdominal circumference [16]. While estimates for each cohort separately differed slightly, they did not reach statistical significance in the Rhea cohort (Greece), which is based in a Mediterranean country [16]. Discrepancies between the Rhea study and our results in the same child age range may probably be attributed to lower education levels, higher smoking rates, and the greater consumption of red and processed meats among women in the Rhea cohort. These maternal risk factors for childhood obesity [39,40] might have attenuated the association. It is worth noting that, unlike the above study, we used multiple FFQs for dietary assessments at different time points, and so our approach provides a more accurate measure of maternal whole-pregnancy diet. Another study based on a Mediterranean population, which used data from the Spanish INMA (INfancia y Medio Ambiente) birth cohort [17], reported an inverse relationship between whole-pregnancy MedDiet and the risk of abdominal obesity but not with overweight/obesity at the age of 4 [17]. Interestingly, a later in-depth analysis with the same INMA cohort [18] that focused on offspring longitudinal BMI trajectories (assessed through repeated BMI measurements at multiple time points during infancy) showed that a higher maternal late-pregnancy MedDiet score was associated with accelerated growth during the first four years from birth, which is linked to the risk of later overweight/obesity [41].

Unlike our results, at least in part, a recent U.S. study from the Nurses’ Health Study II and offspring cohort Growing Up Today Study II [21] reported a protective effect of maternal MedDiet during peripregnancy (a one-year dietary assessment period covering at least part of the pregnancy) on the risk of developing overweight/obesity in adolescents and young adults aged 12 to 23 years. However, this relationship became nonsignificant when the authors further adjusted for maternal confounders such as pre-pregnancy BMI, smoking, and education [21]. It is worth noting that differences in offspring age may limit comparability with our study. Nonetheless, the findings from the aforementioned study suggest that maternal MedDiet during pregnancy may have a diminishing protective effect over time and that offspring overweight/obesity is influenced by multiple maternal behaviors. In our study, the protection against overweight at 4 years of age promoted by the maternal rMedDiet during pregnancy was observed even after adjusting for well-established maternal confounders such as early pregnancy BMI, smoking, and education.

An interesting finding from our stratified analyses is that while whole-pregnancy rMedDiet tended to be inversely associated with childhood overweight/obesity across all subgroups, this association was stronger among women who were under 30 years of age, college-educated, or from a low-middle SES. This underscores the significant influence of maternal sociodemographic and school-level factors on the link between maternal overall diet quality and preschool-aged overweight/obesity, thus highlighting the importance of these factors in shaping preventive policies. It is also important to mention the protective effect of prenatal rMedDiet adherence on childhood overweight/obesity observed in non-smoking mothers but not in smokers. This suggests that smoking during pregnancy could substantially dilute the beneficial effects of maternal rMedDiet. It may be hypothesized that smoking mothers are more susceptible to poor eating habits, thus leading to nutritional deficiencies, or exhibit different health-related behaviors compared to non-smokers, thus potentially influencing their offspring’s unfavorable body composition. In fact, we found that children of smoking mothers were more likely than those of non-smoking mothers to be overweight/obese, which is consistent with prior research [39]. Further investigation is needed to either confirm or refute this tentative hypothesis. Meanwhile, giving up smoking before pregnancy may help to reduce the risk of childhood overweight/obesity.

Maternal obesity and excessive GWG also seem to exert a substantial influence on offspring obesity from childhood to adulthood, which suggests a predominantly genetic predisposition [42]. In line with previous studies [42], we also found that being an overweight/obese mother in early pregnancy or having excessive GWG was associated with overweight/obesity in offspring at age 4. Importantly, our stratified analyses showed that the beneficial impact of maternal rMedDiet on childhood overweight/obesity in 4-year-olds was more prominent in pregnant women who were overweight/obese or who had excessive GWG. As is supported by another groups [43], this suggests that adhering to this healthy dietary pattern during pregnancy may at least partly counteract the genetic predisposition to weight gain.

Our results are also supported by studies of diet quality scores that share similarities with the MedDiet. For example, a recent multi-center study that used data pooled from 16,295 mother–child pairs in seven European birth cohorts within the ALPHABET consortium also reported an inverse association between whole-pregnancy Dietary Approaches to Stop Hypertension (DASH) and overweight/obesity in children aged 10 years [44]. Likewise, in the Mothers and Infants LinKed for Health study, higher diet quality, based on the Healthy Eating Index during pregnancy and lactation, was also associated with lower weight status from birth to 6 months and adiposity at 6 months [45].

Our main emphasis in studying the MedDiet pattern was to conduct a comprehensive assessment of maternal diet quality that encompassed the combined effects of the diet’s foods and nutrients as well as bioactive compounds such as antioxidants and polyphenols. Our previous findings with the same ECLIPSES cohort [14] had revealed that high compliance with this healthy dietary pattern resulted in a more favorable overall nutritional profile among pregnant women. Mothers with a greater adherence to this diet consumed relatively more antioxidant vitamins (vitamins E, C, and beta-carotene), vitamin D, folate, polyunsaturated fatty acid and fiber than those who did not [14]. These factors are believed to reduce susceptibility to childhood weight gain by positively influencing fetal glucose metabolism and fetal metabolic function [46,47] while also supporting a favorable neonatal microbiome [48]. Other studies indicate that maternal deficiency in micronutrients such as vitamin D [49], folate [50], and ω-3 polyunsaturated fatty acids [51] during pregnancy, which is extremely prevalent, has the potential to indirectly impact offspring metabolic programming through epigenetic changes and to ultimately contribute to childhood obesity [35]. Conversely, maternal high-fat (especially saturated fat) diets also result in increased offspring adiposity by modifying DNA methylation and the gene expression of fetal hypothalamic appetite-related neurons and central reward system molecules, thus leading to alterations in appetite and energy metabolism in offspring [51].

Our study has certain limitations. Given its observational nature, a cause–effect relationship cannot be determined, and our results should therefore be interpreted with caution. As this research was conducted on healthy pregnant women who reside in the Mediterranean region and have specific sociodemographic and healthy Mediterranean lifestyle traits, its generalizability is limited. Moreover, like most epidemiological studies, the self-administered FFQ we employed to assess dietary intake focused on the frequency of intake rather than on quantity. However, it is worth noting that our FFQ underwent prior validation within our population. Moreover, we assessed diet at three time points during pregnancy (in the first, second, and third trimesters) and computed an average to capture a more comprehensive representation of the whole-pregnancy diet while also minimizing intra-person variation and measurement errors. This approach can be viewed as advantageous. Our study also possesses other strengths, such as its prospective design with a relatively large sample size as well as adjustments for potential confounding factors and stratified analyses.

## 5. Conclusions

In summary, by using a longitudinal analysis of mothers and their offspring, we have found that closer adherence to the MedDiet during pregnancy may offer protection against the risk of offspring overweight/obesity at 4 years of age. This benefit seems to be even more pronounced in women who are under 30 years old, overweight/obese, non-smokers, or from a lower-middle social class. All in all, given the worldwide extent of the obesity epidemic, our results suggest that adherence to the MedDiet has a potentially significant impact on public health.

## Figures and Tables

**Table 1:** Maternal sociodemographic, lifestyles, and obstetrics characteristics in pregnancy and child characteristics, by weight status of children at 4 years of age.

Characteristics		Total	Normal Weight	Overweight/Obese	*p*-Value
		(n = 272)	(n = 202; 74.3%)	(n = 70; 25.7%)	
Maternal characteristics					
Age (years), mean ± SD		31.6 ± 4.6	31.9 ± 4.3	30.9 ± 5.3	0.129
Age category (years), n (%)					
	<25	21 (8)	11 (5)	10 (14)	
	25–<30	61 (22)	44 (22)	17 (24)	**0.041**
	≥30	190 (70)	147 (73)	43 (61)	
BMI initial (kg/m^2^), mean ± SD		25.1 ± 4.4	24.3 ± 3.9	27.3 ± 5.0	**<0.001**
BMI category (kg/m^2^), n (%)					
	Normal weight (18.5–<25)	158 (58)	132 (65)	26 (37)	
	Overweight (25.0–<30)	76 (28)	50 (25)	26 (37)	**<0.001**
	Obesity (≥30)	38 (14)	20 (10)	18 (26)	
GWG (kg), mean ± SD		10.3 ± 3.6	10.1 ± 3.4	10.5 ± 3.9	0.513
IOM GWG recommendations, n (%) ^†^					
	Adequate	106 (39)	81 (40)	25 (36)	
	Insufficient	112 (41)	93 (46)	19 (27)	**<0.001**
	Excessive	54 (20)	28 (14)	26 (37)	
Educational level, n (%)					
	Low (primary school or less)/ medium (secondary studies)	153 (56)	106 (52)	47 (67)	**0.033**
	High (university or more)	119 (44)	96 (48)	23 (32)	
Familiar SES, n (%)					
	Low/medium	212 (79)	151 (75)	61 (87)	**0.030**
	High	60 (22)	51 (25)	9 (13)	
Smoking status, n (%)		226 (83)	174 (86)	52 (74)	**0.023**
Alcohol consumption in pregnancy, n (%)		20 (8)	12 (7)	8 (13)	0.111
Physical activity (METs/min/week) in pregnancy, mean ± SD		447 ± 664	451 ± 683	436 ± 610	0.868
rMedDiet during pregnancy (points), mean ± SD *		9.8 ± 2.3	10.0 ± 2.3	9.2 ± 2.1	**0.028**
Delivery mode, n (%)					
	Vaginal delivery	216 (79)	163 (81)	53 (76)	0.375
	Cesarean (any)	56 (21)	39 (19)	17 (24)	
Child characteristics					
Sex, female, n (%)		134 (49)	108 (53)	26 (37)	**0.019**
GA at delivery (weeks), mean ± SD		39.7 ± 1.6	39.7 ± 1.5	39.6 ± 1.6	0.621
Age at 4-year visit (years), mean ± SD		4.4 ± 0.4	4.4 ± 0.3	4.4 ± 0.4	0.711
Birth weight (g), mean ± SD		3257 ± 466	3236 ± 440	3317 ± 535	0.217
Weight at 4-year visit (kg), mean ± SD		18.1 ± 3.2	16.9 ± 1.9	21.4 ± 3.9	**<0.001**
Height at 4-year visit (cm), mean ± SD		105.8 ± 5.4	105.4 ± 5.6	107.0 ± 5.1	**0.028**
BMI at 4-year visit (kg/m^2^), mean ± SD		16.0 ± 2.1	15.2 ± 1.0	18.6 ± 2.4	**<0.001**
BMI z-score at 4-year visit, mean ± SD		0.44 ± 1.36	−0.11 ± 0.81	2.08 ± 1.29	**<0.001**

Values are expressed as mean ± SD or number (%). Abbreviations: SD, standard deviation; BMI, body mass index; GWG, gestational weight gain; IOM, Institute of Medicine; METs, metabolic equivalent of task, rMedDiet, Mediterranean diet; GA, gestational age. † Recommendations for GWG from the IOM guidelines are for initial BMI < 18.5 kg/m^2^, total weight gain 12.5–18 kg; for BMI between 18.5 and 24.9 kg/m^2^, total weight gain 11.5–16 kg; for BMI between 25.0 and 29.9 kg/m^2^, total weight gain 7–11.5 kg; and for BMI ≥30 kg/m^2^, total weight gain between 5 and 9 kg. * n = 252. *p*-values were calculated with the chi-square test or Student’s *t*-test. The significance of the numbers in bold is *p*-value < 0.05.

**Table 2:** Odds ratios (ORs) and 95% confidence intervals (95% CIs) for associations between maternal adherence to the rMedDiet (continuous and quartiles) during pregnancy and childhood overweight/obesity at 4 years of age.

rMedDiet Adherence Score (Point)		N Total/ Cases (%)	Univariable Model	*p*-Value	Multivariable Model	*p*-Value
			OR (95% CI)		OR (95% CI)	
Continuous (per 1-point increase)		252/62 (24.6)	0.86 (0.75–0.98)	**0.030**	0.81 (0.68–0.95)	**0.010**
Quartiles of adherence to the rMedDiet						
	Q1 (≤8 points)	73/22 (30.1)	1 (ref.)		1 (ref.)	
	Q2 (9–10 points)	91/23 (25.3)	0.78 (0.39–1.56)	0.488	0.76 (0.34–1.69)	0.501
	Q3 (11 points)	31/7 (22.6)	0.67 (0.25–1.80)	0.433	0.53 (0.17–1.65)	0.279
	Q4 (≥12 points)	57/10 (17.5)	0.49 (0.21–1.15)	0.102	0.34 (0.12–0.90)	**0.038**
	*p* for trend		0.098		**0.037**	

Logistic regression models were used to calculate Odds Ratios (OR) and 95% confidence intervals (IC al 95%). The multivariable logistic regression model was mutually adjusted for age (<25 (ref.), 25–29, ≥30 years); initial BMI (normal (BMI < 25 kg/m^2^) (ref.), overweight (BMI 25–29.9 kg/m^2^), obesity (BMI ≥ 30 kg/m^2^)); weight gain during pregnancy (adequate (ref.), insufficient, excessive); education level (low/medium (primary/secondary) (ref.), high (university students)); SES (low/medium (ref.), high); smoking status (no (ref.), yes), mode of delivery (normal vaginal (ref.), cesarean (any)); gestational age at delivery (weeks); sex of the child (male, female); birth weight (g); and age of the child (years) at the 4-year visit. The significance of the numbers in bold is *p*-value < 0.05. Abbreviations: rMedDiet, Mediterranean diet; Q, quartile.

## Data Availability

The datasets generated and/or analyzed during the current study are not publicly available due to subject confidentiality but are available from the corresponding author on reasonable request.
